# Mapping the Evolution of Scientific Fields

**DOI:** 10.1371/journal.pone.0010355

**Published:** 2010-05-04

**Authors:** Mark Herrera, David C. Roberts, Natali Gulbahce

**Affiliations:** 1 Department of Physics and Institute for Research in Electronics and Applied Physics, University of Maryland, College Park, Maryland, United States of America; 2 Theoretical Division and Center for Nonlinear Studies, Los Alamos National Laboratory, Los Alamos, New Mexico, United States of America; 3 Department of Physics and Center for Complex Networks Research, Northeastern University, Boston, Massachusetts, United States of America; 4 Center for Cancer Systems Biology, Dana Farber Cancer Institute, Boston, Massachusetts, United States of America; Indiana University - Bloomington, United States of America

## Abstract

Despite the apparent cross-disciplinary interactions among scientific fields, a formal description of their evolution is lacking. Here we describe a novel approach to study the dynamics and evolution of scientific fields using a network-based analysis. We build an *idea* network consisting of American Physical Society Physics and Astronomy Classification Scheme (PACS) numbers as nodes representing scientific concepts. Two PACS numbers are linked if there exist publications that reference them simultaneously. We locate scientific fields using a community finding algorithm, and describe the time evolution of these fields over the course of 1985–2006. The communities we identify map to known scientific fields, and their age depends on their size and activity. We expect our approach to quantifying the evolution of ideas to be relevant for making predictions about the future of science and thus help to guide its development.

## Introduction

Cross-fertilization between different scientific fields has been recognized for its ability to encourage new developments and innovative thinking. For this reason, multidisciplinary approaches to research are becoming more popular. Some recent examples include applying physics techniques to the study of biological phenomena [1], deriving an understanding of the nature of critical phenomena from renormalization techniques in particle physics [2] drawing inferences about the early universe from findings in terrestrial superfluid experiments [3], and using statistical physics to analyze technological and social systems [4].

In an effort to move beyond anecdotal evidence of the benefit of interdisciplinary discourse for science, in this paper we study the dynamics of groups, or “communities”, of ideas using a statistical physics approach. We attempt to quantify the evolution of ideas and subdisciplines within physics as they emerge, interact, merge, stagnate, and desist. The quest for describing the development of scientific fields is not new. There have been epidemiological [5], [6] and network-based approaches (citation and collaboration networks) [7]–[15] aiming to gain insight into the spread of scientific ideas. Recently the temporal evolution of several scientific disciplines have been modeled with a coarse-grained approach [16].

Here we build a scientific concept network consisting of American Physical Society PACS numbers as nodes representing scientific concepts. The American Institute of Physics (AIP) develops and maintains the PACS scheme as a service to the physics community in aiding the classification of scientific literature and information retrieval. Two PACS numbers are linked if there exist publications that reference them simultaneously. Our approach differs from previous methods in that it provides a direct, unsupervised description of scientific fields and uses techniques such as community finding and tracking from the field of network physics. This approach provides means to quantify how ideas and movements in science appear and fade away. Because this method makes it possible to measure the current and past state of the relationship between scientific concepts, it may also help to make predictions about the future of science and thus inform efforts to guide its development. In this paper, we entertain some of the quantitative questions that this method permits; specifically, we seek to answer questions about the relationship between size, lifetime, and activity of scientific fields.

Various local to global topological measures have been introduced to unveil the organizational principles of complex networks [17]–[19]. One such measure that allows the discovery of organizational principles of networks is community finding. There have been a number of methods to find the communities in networks which describe the inherent structure or functional units of a network [20]–[23]. One of these is CFinder, a clique percolation method (CPM) introduced by Palla et al. [21], which finds overlapping communities and is especially suitable for studying the evolution of scientific fields since scientific concepts are often shared among multiple fields. We use this CPM to track the evolution of physics.

## Results

### Building the Network

Data were collected from the American Physical Society's (APS) *Physical Review* database from 1977–2007. Journals included in the study are *Physical Review Letters*, *Physical Review {A* through *E}*, and *Physical Review Special Topics: Accelerators and Beams*. Papers in this database contain a list of author-assigned PACS codes, where each PACS code refers to a specific topic in physics. PACS itself is hierarchical, which is evident in the structure of the codes with up to 5 levels of topic specification. For example the PACS code ‘64.60.aq’ has 5 levels where the first digit ‘6’ represents the first level (in this case ‘condensed matter’), ‘4’ represents the second (e.g. ‘equations of state, phase equilibria, and phase transitions’), the third and fourth digits ‘60’ together represent the third level (e.g., ‘general studies of phase transitions’) while the last two characters ‘aq’ carry information pertaining to the fourth and fifth levels of specification (e.g. ‘specific approaches applied to phase transitions’ and ‘networks’, respectively).

PACS codes are not static, rather, the coding scheme is periodically updated with the addition and deletion of codes. In order to (at least partially) account for this effect, the scientific concept network was constructed such that the nodes in the network represent individual PACS codes using the first four digits of specification, where changes to scheme are less probable. This network and the related material is available on our website [24]. In our network, an *edge* occurs between two nodes if the two PACS codes they represent are cited in the same paper; one paper in the database often contributes many nodes and edges to the network. Furthermore, edges are weighted by the number of papers that contain that edge. We introduce two measures, node and edge cutoffs, to control for noise in the network (see Methods section).

**Figure 1:**
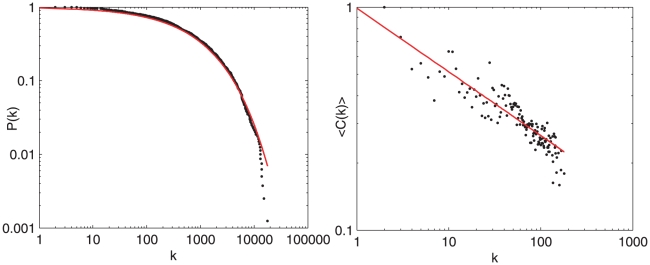
Measurements on the PACS network from 1977–2007. A) Cumulative degree distribution P(k) of the PACS network. The red line is a fit to the data. Both the weighted and unweighted cases follow a stretched exponential distribution. B) **Average clustering vs degree for the PACS network, demonstrating that** 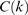 **has some dependence on degree.** Thus, there is some hierarchical structure present in the network.

The entire PACS network from 1977–2007 after both noise measurements were applied has 803 nodes and 23707 distinct edges. The *degree* of a node is the number of edges shared by the node. The weighted cumulative degree distribution follows a stretched exponential with the form, 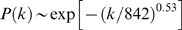 as shown in Fig. 1A. The distribution has a similar form in the unweighted case. The dynamic classification scheme of the American Physical Society, implemented by the addition, splitting and removal of codes, may be preventing the formation of large hubs, thus keeping the specification of the codes more useful. The stretched exponential distribution may be the result of a sublinear-linear attachment type growth [25].

The PACS network also exhibits a weak but apparent hierarchical structure measured by the dependence of the *clustering coefficient* on (unweighted) degree. For a node 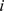, the clustering coefficient is given as 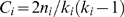, where 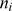 is the number of edges that link the neighbors of node 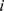, and 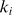 is the degree of the node. The clustering coefficient for a node is the ratio of the number of triangles through node 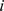 over the possible number of triangles that could pass through node 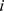 [26]. A purely hierarchical network will have a 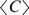 that scales as a power of 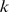, 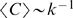, while a random network will have a clustering coefficient that is constant with 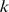 [26]. For this network, 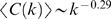, shown in Fig. 1B. This dependence is not surprising given the hierarchical structure of the classification scheme.

### Defining Communities in Physics

Papers published between 1985 and 2006 were used to study the community evolution of the network; 1985 appears to be the first year when all journals present (*Physical Review E* began publication in 1993) consistently used the PACS data scheme, and 2007 was thrown out to exclude incomplete data from the analysis. The journal *Physical Review Special Topics: Accelerators and Beams* was not included because of an irregular publishing schedule. After the noise measures were carried out, the edge weights were no longer used, and the network became an unweighted network with respect to the community evolution analysis. The data were organized into 44 time bins, with each bin representing a 0.5 year time period. Once a paper (and the edges and nodes it contains) appears in the analysis, it is assigned a lifetime, 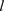, of 0 or 2.5 years. This assignment is an attempt to more realistically capture the nature of scientific dissemination, as well as the delay in time from publication to assimilation by the field. The analysis of community evolution begins at the time bin subsequent to the lapse of the assigned lifetime. Thus the first time bin, 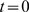, for a paper lifetime of 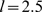 refers to the latter half of 1987 since we start the analysis in 1985.

**Figure 2:**
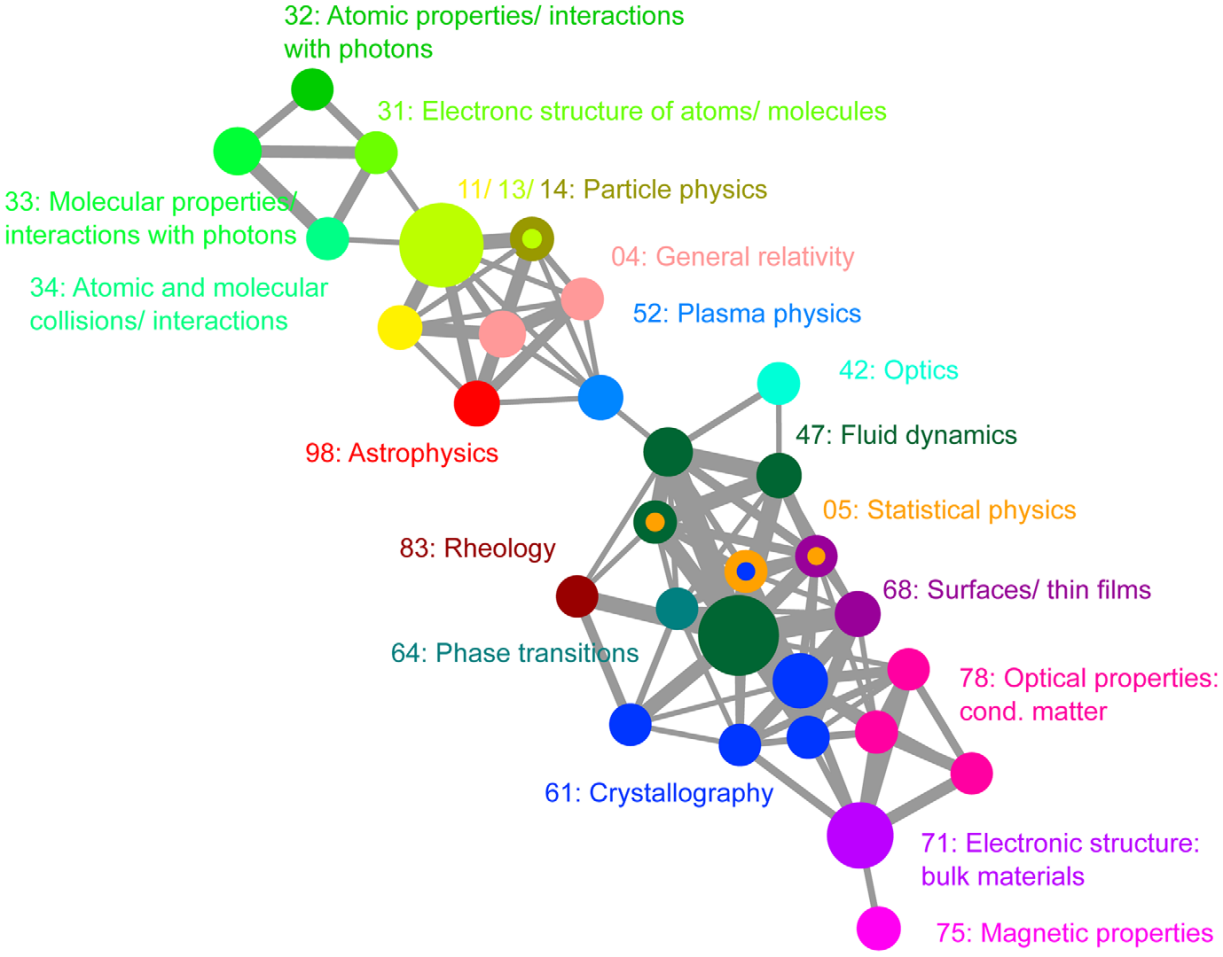
The scientific concept network for the first half of 1997. Nodes corresponding to scientific fields, as well as node labels and their corresponding fields, are shown in the same color. The size of the nodes corresponds to the number of PACS codes contained in that community. Same-color neighboring nodes have the same label. The thickness of the edges correspond to the number of shared PACS codes between communities (the weight of the edge). The community structure is shown at 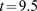 years, corresponding to first half of 1997, using CFinder with 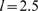 years. Labels are assigned by looking at the first two digits of the PACS codes that make up the largest fraction of each community.

**Figure 3:**
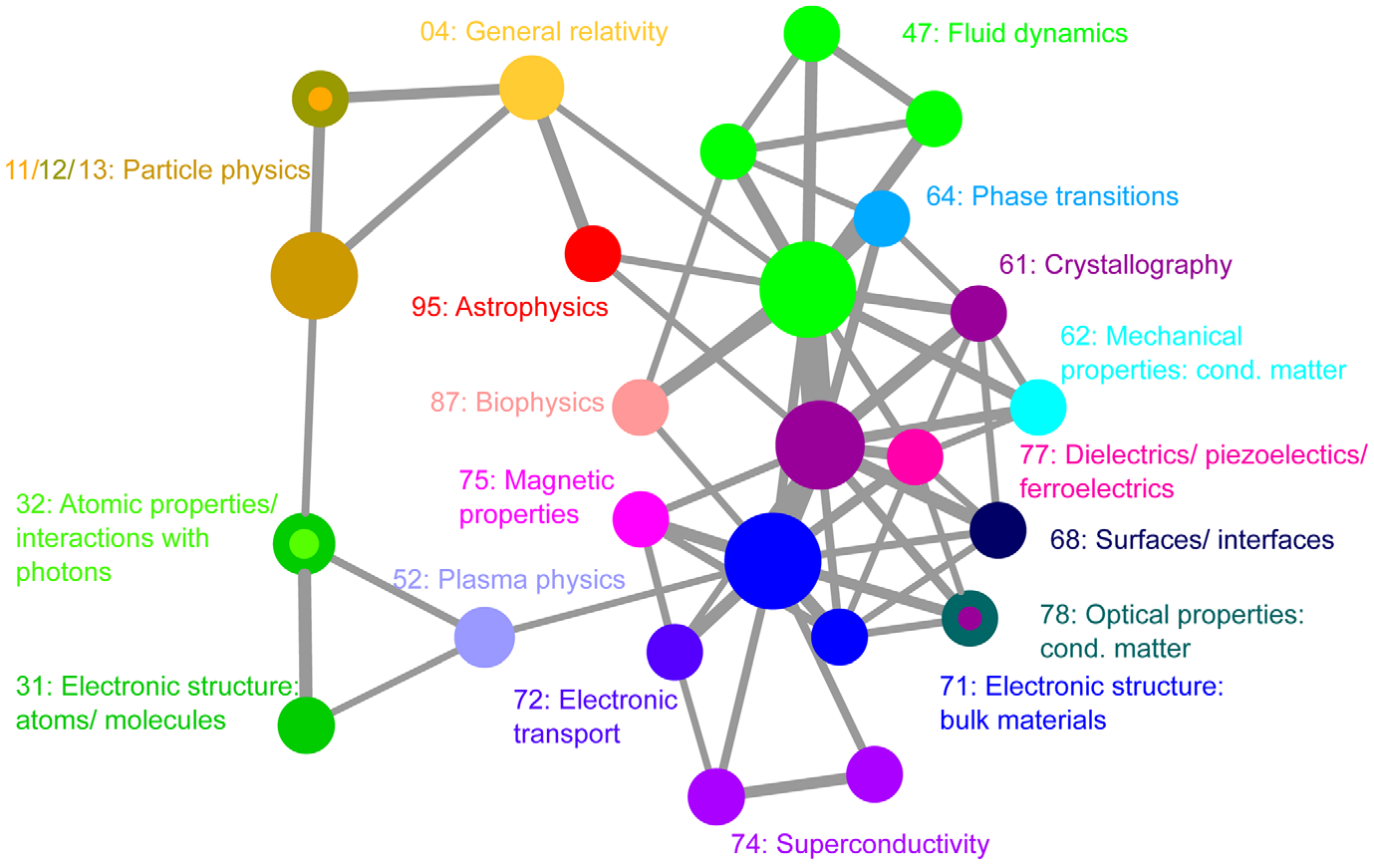
The scientific concept network for the first half of 2005.

In order to study the evolution of different fields in physics, one must first find these fields in our network. We hypothesize that scientific fields are represented by communities in our PACS network. These communities are found using the CFinder algorithm, which is based on a clique percolation method [21]. Figs. 2 and 3 present examples of the community structure extracted utilizing CFinder.

For each community, the code (using only the first two digits) that encompasses the largest fraction of nodes in the community was found. Its name, specified by the PACS scheme, is then used to label the community. If a community has multiple codes which compose the same largest fraction of nodes in that community, then the community is assigned multiple labels. As shown in Figs. 2 and 3, we observe that the analysis captures expected scientific connections among fields in physics. For example, in 1997, particle physics is linked to both general relativity and astrophysics. It is also worthwhile to note the emergence of biophysics as a community in the 2005 analysis.

### Community Evolution and Dynamics

In order to track the evolution of scientific fields, after identifying communities at each individual time interval, it is necessary to match the communities between adjacent time steps. We implemented a community evolution algorithm developed by Palla et al. [27] to match the communities between time bins (see Methods section).

To gain a better understanding of the dynamics of evolving communities, we defined two properties of each community: size and activity. A value for each of these measures can be assigned to every community for each individual time bin. The size 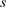 of a community is the number of nodes contained within that community at time 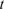. Size can be interpreted as a measure of a community's breadth: communities with a small size encompass only a few distinct ideas, while large communities encompass many distinct ideas. (The cumulative size distribution was calculated for different times and is displayed in Fig. S1.)

The activity 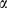 of a community is defined as the number of papers that contain at least one node from that community at time 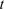. As one expects, there is a strong correlation between size and activity (see Fig. S2).

**Figure 4:**
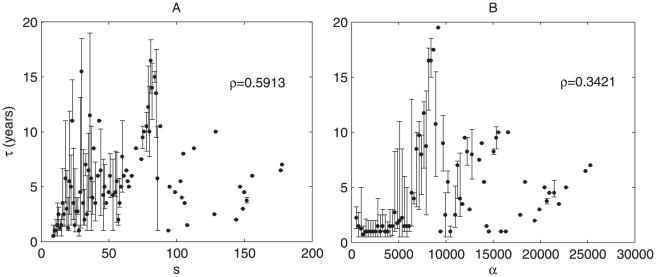
Lifetime measurements of PACS communities. For 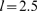 years, the median lifetime (years) as a function of A) size; B) activity 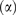. Error bars represent the 1st and 3rd quartiles respectively. For both sets of data, the Spearman's rank correlation coefficient, 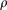, was computed using the unbinned data.

Next, we study the relationship between the age or lifetime of a community versus its size and activity. The age of a community at time 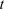 is simply the number of time bins the community has been present in the evolution analysis: 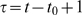, where 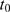 is the time bin in which the community was born. In order to study the dependence of age on size, in each time bin, the current age 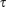 and size 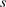 are recorded. Using all communities from all time intervals, the median age is calculated for communities with the same size as shown in Fig. 4A. There is a trend of 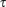 increasing with size 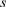. Thus, it would appear that older communities tend to contain more nodes, and that longer lived fields tend to encompass many distinct ideas. Values for both the Pearson correlation coefficient, 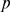, and the Spearman's rank correlation coefficient, 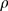, were calculated between 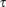 and 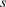 using the raw, unbinned data. 
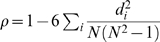
 where 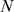 is the number of data points and 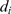 is the difference in the statistical rank of the corresponding values for each data point. For 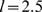, the Pearson correlation coefficient was 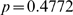 while the Spearman's 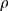 was calculated to be 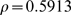.

In order to measure the dependence of age on activity, the current age 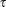 is recorded along with the current activity 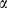 of every community in each time step. Because of the wide range of possible values for activity and noise in the data, the values of 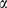 are sorted into 100 equally sized bins. The median age is calculated for all communities within the same activity interval. There is a trend of 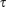 increasing with activity as shown in Fig. 4B which can be partially understood by the strong correlation between size and activity. Further we note an apparent phase transition in activity; as shown in Fig. 4B after some critical value, communities tend to be longer lived. This transition also appears for 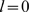 (see Fig. S3). Lifetime as a function of size, 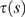, for 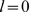 is shown in Fig. S4. Again, the Pearson correlation coefficient and the Spearman's rank correlation coefficient were calculated for 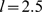 using the raw, unbinned data between 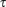 and 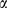, with 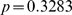 and 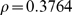.

## Discussion

In this paper, we have developed an approach that enables the quantitative study of the evolution of physics fields, specifically by following the dynamical connections between various ideas within physics. From our investigation, we have shown that long lived communities tend to be larger, and are associated with a higher number of papers.

Our approach opens up an interesting possibility of being able to predict community dynamics and impact from the current network structure. Furthermore, this method can be easily adapted to other scientific fields using different databases. One such is the INSPEC database which has comprehensive coverage of research activity in computer science and engineering in addition to physics, and has an expert-assigned classification scheme rather than author-based assignments.

## Materials and Methods

### Noise Measures

A node cutoff is introduced such that in a given time interval a node must appear at least twice to be included in the network. This measure eliminates many of the typographical errors occurring in the database. The edge cutoff, however, takes into account the random expectation of two PACS codes co-occurring in the same paper. For this cutoff, the weight of an edge between nodes 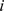 and 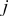, 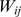, which is the number of papers that both codes 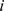 and 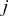 appear in, is compared to the weight expected at random, 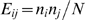, where 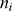 and 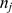 are the number of papers containing nodes 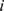 and 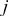 respectively, and 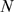 is the total number of papers present in the time interval. If 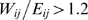, then the appearance of the edge is significant compared to random appearance, and we include it in the network.

### CFinder

The CFinder algorithm is described in detail in Ref. [21]. A community is defined as a union of all 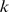-cliques (complete subgraphs of size 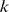) that can be reached from each other through a series of adjacent 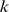-cliques (where adjacency means sharing 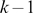 nodes) [21].

### Picking a 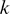 value

For this study, 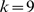 was principally used (for l = 2.5) because it appears to produce a large number of communities while discouraging the formation of giant communities. Further, by keeping 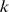 constant, we keep the resolution constant for the entire analysis. Picking an appropriate 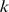 value for the analysis is done by considering two properties: the number of communities present, and the presence of overly large communities [21]. It is desirable to have a large number of communities, so as to increase the statistical quality of measurements made on the network. Fig. S6 plots the number of present communities for each time step for 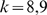, and 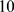, for 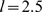. As demonstrated, the number of communities found using the choice of 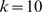 tends to be less than the other parameter choices, making it less favorable in terms of improving statistical quality.

A 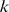 value must also be large enough to avoid the introduction of overly large communities that obscure the actual community structure of the network [21]. To quantify this property, we use the quantity 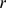 which is the ratio of the size of the largest community to the second largest community for a given time bin. Thus while some distribution in the sizes of communities is necessary, 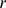 should not be overly large. Fig. S7 plots the measure 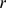 against all time bins for 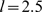. For 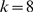, the values of 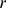 tends to be larger than (signifying giant communities) than those calculated from the other two parameter values, making it an unfavorable parameter choice.

### Community Matching

The community matching algorithm is described in detail in Ref. [27]. In this analysis, an appropriate 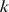-value is used rather than a constant edge-weight cutoff. A running stationarity measure is described in Appendix S1 and Figure S5. The merger of two communities is described in Appendix S1 and Figures S8 and S9.

## Supporting Information

Appendix S1 An appendix containing descriptions of the supporting information and figures.
(0.06 MB PDF)

Figure S1 The cumulative size distribution for various times in the network. The distributions appear long tailed over one decade.
(0.03 MB EPS)

Figure S2 The activity α of each community plotted against its size s for every time interval (l = 2.5). Notice the positive correlation between α and s.
(0.02 MB EPS)

Figure S3 The median lifetime as a function of activity for k = 7, l = 0. Notice the trend of τ increasing with activity.
(0.01 MB EPS)

Figure S4 The median lifetime as a function of size for k = 7, l = 0. Notice the trend of τ increasing with size.
(0.02 MB EPS)

Figure S5 Age of each community (k = 7, l = 0) vs. its running stationarity value for all time bins.
(0.02 MB EPS)

Figure S6 The number of communities present in the network (after the noise measures have been applied) as a function of time for various k values, with l = 2.5. In order to improve the statistical quality of the analysis, larger numbers of communities are favorable, making k = 10 an unfavorable parameter choice.
(0.01 MB EPS)

Figure S7 The ratio r of the size of the largest community present divided by size of the second largest community for every time bin for l = 2.5. Large r indicates the presence of overly large communities that obscure the community structure; thus k = 8 is an unfavorable choice of parameter.
(0.01 MB EPS)

Figure S8 Size of the nuclear physics community vs time for k = 9 and k = 10, using l = 2.5. While the community appears to die at t = 8 (4 years) for k = 9, a community of similar nodes is seen to continue beyond the time of apparent death when using the higher community cohesiveness requirement of k = 10. It is possible then that the nuclear physics community is still present in the analysis, but has merged with another community.
(0.01 MB EPS)

Figure S9 Merger of the nuclear physics community (green) with another community (particle physics: specific reactions and phenomenology) at the time of apparent death, t = 8 (4 years) for the nuclear physics community
(0.51 MB EPS)
