# Supplementary material for: Mapping the Evolution of Scientific Fields
Source: PLoS One. 2010 May 4;5(5):e10355. doi: 10.1371/journal.pone.0010355 (PMC2864309; doi:10.1371/journal.pone.0010355)
Supplement: Appendix S1 — An appendix containing descriptions of the supporting information and figures. (0.06 MB PDF) [file pone.0010355.s001.pdf]

S1: Appendix

Mapping the Evolution of Scientific Fields

Mark Herrera[1], David C. Roberts[2,*], Natali Gulbahce [3,*]
1 Department of Physics and Institute for Research in Electronics and Applied Physics, University of Maryland, College Park, MD, USA
2 Theoretical Division and Center for Nonlinear Studies, Los Alamos National Laboratory, Los Alamos, USA
3 Department of Physics and Center for Complex Networks Research, Northeastern University, Boston, MA, USA
Center for Cancer Systems Biology, Dana Farber Cancer Institute, Boston, MA
∗ **E-mail: dcr@lanl.gov, natali.gulbahce@gmail.com**

1 Community Dynamics

Once community structure is established, a variety of different measurements are performed on the dynamics of the evolving communities.

The cumulative community size distribution appears long tailed over one decade, which is robust as a function of t (years), as shown in Fig. S1.

Fig. S2 plots for all time intervals the size of every community against its activity α for paper lifetime $l = 2.5$. There appears to be a positive correlation between the two measures, and this trend is observed for $l = 0$ (not shown).

The dependence of age on activity was measured and Fig. S3 shows the results for the τ vs. α measurements for $k = 7$ with a paper lifetime of $l = 0$ years, where α values were binned because of the wide range in α, as well as to reduce noise. In both cases (the one presented here and the one presented in the letter) there is a trend of age increasing as activity increases (though less apparently for $l = 0$) and, as expected given the correlation between α and s, one sees a similar relationship between age and size, as shown in Fig. S4. Thus, older communities also tend to encompass more publications, a result that agrees with naive expectation. Further, we note an apparent phase transition in both paper lifetime cases (more apparent for l=2.5 than for $l = 0$); after some critical α, communities tend to be longer lived.

Further understanding of the community dynamics can be gained by studying the volatility of the evolving communities, a measure of how much communities tend to change between subsequent time steps. To see this, we define an *age dependent* running stationarity $\xi(\tau)$ based on community correlation and stationarity presented by Palla et al. [1]. The correlation $C(t, t')$ between two states of the same community $A(t)$ at times t and t' is

$$C(t, t') = \left| \frac{A(t) \cap A(t')}{A(t) \cup A(t')} \right|.$$

Then the running stationarity, $\xi(\tau)$, of that community is the average correlation between subsequent

time steps up to age τ,

$$\xi(\tau) = \frac{1}{\tau - 1} \sum_{t'=t_0}^{t_0+\tau-2} C(t', t'+1).$$

The running stationarity, $\xi(t)$, is plotted against lifetime for every community with $\tau > 1$ along with its current age at time t, for all t, for $l = 0$ in Fig. S5. This result is qualitatively similar to results obtained using randomized correlations. For larger values of l, the distribution shifts to larger values of $\xi(t)$.

2 Picking a k value

Throughout the paper, $k = 9$ is principally used (for l=2.5) because it appears to produce a large number of communities while discouraging the formation of giant communities. Further, by keeping k constant, we keep the resolution constant for the entire analysis. Picking an appropriate k value for the analysis is done by considering two properties: the number of communities present, and the presence of overly large communities [2]. It is desirable to have a large number of communities, so as to increase the statistical quality of measurements made on the network. Fig. S6 plots the number of present communities for each time step for $k = 8, 9$, and 10, for $l = 2.5$. As demonstrated, the number of communities found using the choice of $k = 10$ tends to be less than the other parameter choices, making it less favorable in terms of improving statistical quality.

A k value must also be large enough to avoid the introduction of overly large communities that obscure the actual community structure of the network [2]. To quantify this property, we use the quantity r which is the ratio of the size of the largest community to the second largest community for a given time bin. Thus while some distribution in the sizes of communities is necessary, r should not be overly large. Fig. S7 plots the measure r against all time bins for $l = 2.5$. For $k = 8$, the values of r tend to become larger (signifying giant communities) than those calculated from the other two parameter values, making it an unfavorable parameter choice.

3 Merging of communities

Lastly, we present an example of a merger between two communities. Tracking a nuclear physics community, Fig. S8 shows the size of that community as a function of time for $k = 9$ and a community of similar nodes with $k = 10$, using $l = 2.5$.

With $k = 9$, it appears that this particle physics community abruptly dies at $t = 4$ years. Increasing the cohesiveness of communities by increasing to $k = 10$ demonstrates that a community composed of similar nodes continues to propagate past this time of apparent death. Thus, it seems that the nuclear physics community is still present in the network, but has become absorbed by another community.

Fig. S9 plots a community at $t = 4$ years with the nodes from the nuclear physics community displayed in green. We can assign a label to this community in the usual manner using the nodes present just before the apparent death of the nuclear physics community. Doing so, the absorbing community is comprised of the ‘physics of elementary particles and fields: specific reactions and phenomenology’ in the time bin prior to its absorption of the particle physics community.

References

1. Palla G, Barabasi AL, Vicsek T (2007) Quantifying social group evolution. Nature 446: 664–667.

2. Palla G, Derenyi I, Farkas I, Vicsek T (2005) Uncovering the overlapping community structure of complex networks in nature and society. Nature 435: 814–818.

Figure Legends

Figure 1. The cumulative size distribution for various times in the network. The distributions appear long tailed over one decade.

Figure 2. The activity α of each community plotted against its size s for every time interval ($l = 2.5$). Notice the positive correlation between α and s.

Figure 3. The median lifetime as a function of activity for $k = 7$, $l = 0$. Notice the trend of τ increasing with activity.

Figure 4. The median lifetime as a function of size for $k = 7$, $l = 0$. Notice the trend of τ increasing with size.

Figure 5. Age of each community ($k = 7$, $l = 0$) vs its running stationarity value for all time bins.

Figure 6. The number of communities present in the network (after the noise measures have been applied) as a function of time for various k values, with $l = 2.5$. In order to improve the statistical quality of the analysis, larger numbers of communities are favorable, making $k = 10$ an unfavorable parameter choice.

Figure 7. The ratio r of the size of the largest community present divided by size of the second largest community for every time bin for $l = 2.5$. Large r indicates the presence of overly large communities that obscure the community structure; thus $k = 8$ is an unfavorable choice of parameter.

Figure 8. Size of the nuclear physics community vs time for $k = 9$ and $k = 10$, using $l = 2.5$. While the community appears to die at $t = 8$ (4 years) for $k = 9$, a community of similar nodes is seen to continue beyond the time of apparent death when using the higher community cohesiveness requirement of $k = 10$. It is possible then that the nuclear physics community is still present in the analysis, but has merged with another community.

Figure 9. Merger of the nuclear physics community (green) with another community (particle physics: specific reactions and phenomenology) at the time of apparent death, $t = 8$ (4 years) for the nuclear physics community.
